# Characteristics of major and macular branch retinal vein occlusion

**DOI:** 10.1038/s41598-022-18414-2

**Published:** 2022-08-18

**Authors:** Yu-Jin Choi, Donghyun Jee, Jin-woo Kwon

**Affiliations:** 1 Department of Ophthalmology and Visual Science,College of Medicine, The Catholic University of Korea, Seoul, Korea; 2 Department of Ophthalmology and Visual Science, St. Vincent’s Hospital, Jungbu-daero 93, Paldal-gu, Suwon, 16247 Korea

**Keywords:** Predictive markers, Outcomes research, Eye diseases

## Abstract

We compared the aqueous profiles, baseline characteristics, and clinical outcomes of 54 eyes with macular edema secondary to major branch retinal vein occlusion (BRVO) and macular BRVO. We also identified the characteristics of poor responders to anti-vascular endothelial growth factor (VEGF) injections. Aqueous inflammatory cytokine and VEGF concentrations were significantly higher in major BRVO. In optical coherence tomography, major BRVO had a higher proportion with subretinal fluid, disorganization of retinal inner layers, and ellipsoid zone disruption. Comparing the clinical outcomes, major BRVO required more intravitreal anti-VEGF injections and had a poorer visual prognosis in the first 12 months. A significantly higher proportion of patients with major BRVO required additional treatments after 6 months compared to macular BRVO. Patients who responded poorly to anti-VEGF had higher aqueous VEGF levels and central subfield thickness (CST) at baseline. In conclusion, major BRVO patients required more and longer treatments, and had worse visual prognoses. BRVO that responds poorly to anti-VEGF had greater CST and higher aqueous VEGF levels at baseline.

## Introduction

Retinal vein occlusion (RVO) is a common retinal vascular disease, with a prevalence of 0.5–2.0%^1,2^. It can cause permanent visual disturbance by itself or by complications such as macular edema (ME), neovascular glaucoma, and vitreous hemorrhage^1^.

An early diagnosis and intervention are important to keep visual function and to obtain good clinical results. RVO is divided into central and branch RVO (BRVO) and BRVO is subdivided into major and macular BRVO according to the anatomical location of occlusion^3^. BRVO results from venous occlusion of any branch of the central retinal vein. While major BRVO refers to occlusion of a retinal vein that drains one of the quadrants, macular BRVO refers to occlusion of a venule within the macula^4^.

ME is a common cause of visual disturbance in patients with BRVO and involves inflammatory cells, cytokines, growth factors, and enzymes^5^. Anti-VEGF and steroid agents are used to treat BRVO with ME, and have good anatomic and visual outcomes^6,7^. However, the response to these agents varies from patient to patient. Several studies have sought to predict the prognosis or responsiveness to various treatments, using optical coherence tomography (OCT), OCT angiography (OCTA), and biomarkers in the ocular fluid, or systemic evaluation^8,9^. However, there is no consensus on predictors of the prognosis of BRVO with ME. Therefore, we examined biomarkers and factors associated with the prognosis of anti-VEGF treatments of patients with ME secondary to BRVO.

## Results

We enrolled 54 BRVO with ME eyes of 54 patients with a mean age 65.04 ± 11.66 years (17 males and 37 females). The average time interval between onset of symptoms and treatments was 17.81 ± 14.33 days. At baseline, the mean best corrected visual acuity (BCVA) (LogMAR) was 0.53 ± 0.33 and the mean central subfield thickness (CST) was 455.09 ± 14.04 µm.

When classified by anatomical location 35 (64.81%) eyes had major BRVO and 19 (35.19%) had macular BRVO. The systemic and ocular characteristics of all patients are summarized in Table 1 according to this classification.

**Table 1:** Demographics and clinical characteristics of major and macular BRVO patients at baseline.

			Macular BRVO (n = 19)	Major BRVO (n = 35)	*P*
Systemic factors	Sex (male:female)		8:11	9:26	0.216
	Age (years)		62.0 (56.0;70.5)	61.0 (58.0;76.5)	0.663
	Diabetes mellitus		7 (36.84%)	11 (31.43%)	0.687
	Hypertension		10 (52.63%)	22 (62.86%)	0.465
	Dyslipidemia		3 (15.79%)	13 (37.14%)	0.101
Aqueous humor cytokines (pg/mL)	IL-1β		0.00 (0.00, 0.00)	0.00 (0.00, 0.00)	0.061
	IL-2		0.00 (0.00, 2.93)	2.24 (0.00, 9.25)	0.094
	IL-6		3.40 (1.50, 4.58)	11.01 (5.52, 15.53)	< 0.001
	IL-8		8.76 (6.79, 13.34)	33.75 (22.15, 65.34)	< 0.001
	IL-10		0.28 (0.00, 0.92)	0.88 (0.54, 1.41)	0.003
	IL-17		0.00 (0.00, 1.21)	0.00 (0.00, 1.30)	0.802
	TNF-α		2.20 (1.87, 3.48)	4.79 (3.28, 6.11)	< 0.001
	VEGF		30.72 (22.04, 43.31)	71.56 (51.00, 112.58)	< 0.001
	PlGF		2.31 (1.29, 3.31)	3.54 (2.65, 4.71)	0.007
Ocular factors	Baseline CST (µm)		365.0 (332.0, 419.0)	447.0 (334.0, 584.0)	0.145
	Baseline SFCT (µm)		257.95 ± 31.09	269.54 ± 39.54	0.274
	Baseline SFCT of contralateral eye (µm)		223.00 [204.00;232.00]	219.00 [207.00;237.00]	0.842
	Baseline BCVA (LogMAR)		0.40 (0.20, 0.55)	0.30 (0.20, 0.50)	0.358
	Subretinal fluid		5 (26.32%)	21 (60.00%)	0.018
OCT findings	EZD grade	0	11 (57.89%)	16 (45.71%)	0.019
		1	8 (42.11%)	8 (22.86%)	
		2	0 (0%)	11 (31.43%)	
	DRIL		5 (26.32%)	21 (60.00%)	0.037

Values are expressed as the mean ± SD or median and interquartile range, as appropriate.

BRVO, branch retinal vein occlusion; SRF, subretinal fluid; IL, interleukin; TNF, tumor necrosis factor; VEGF, vascular endothelial growth factor; PlGF, placental growth factor; CST, central subfield thickness; SFCT, sub-foveal choroidal thickness; BCVA, best-corrected visual acuity; EZD, ellipsoid zone disruption; DRIL Disorganization of retinal inner layers.

The aqueous levels of interleukin (IL)-6, IL-8, IL-10, tumor necrosis factor (TNF)-α, VEGF, and placental growth factor (PlGF) were significantly higher in the major BRVO group than in the macular BRVO group (*P* < 0.001, *P* < 0.001, *P* = 0.002, *P* = 0.001, *P* < 0.001, and *P* = 0.003, respectively). Subretinal fluid and DRIL (Disorganization of retinal inner layers) in OCT was found in a higher proportion of major BRVO (*P* = 0.009 and* P* = 0.037, respectively). Post hoc analyses showed the proportions of grades 2 to grade 0 in ellipsoid zone disruption (EZD) and grade 2 to grade 1 in EZD were significantly higher in major BRVO compared to macular BRVO (*P* = 0.012 and *P* = 0.005, respectively).

Of the 54 patients, six did not respond to three consecutive Intravitreal bevacizumab (IVB) monthly treatments and were given intravitreal dexamethasone implants. Analyzing the 48 patients who received IVB as needed, the CST was 439.27 ± 140.04 µm at baseline and decreased to 255.56 ± 34.62 at 12 months; the logMAR BCVA was 0.53 ± 0.34 at baseline and improved to 0.43 ± 0.34 at 12 months Comparing the two groups, major BRVO required more IVB treatments in 12 months (3.03 ± 1.16 vs. 2.28 ± 1.18, *P* = 0.045) and had a worse logMAR BCVA at 12 months (0.51 ± 0.37 vs. 0.29 ± 0.21, *P* = 0.035). More eyes required additional treatment after 6 months in major BRVO (*P* = 0.024). However, there were no significant differences in CST at 12 months (Table 2).

**Table 2:** Outcomes of BRVO treatment depending on the anatomical location.

		Macular BRVO (n = 18)	Major BRVO (n = 30)	*P*
CST (µm)	At baseline	360.0 (328.0, 405.0)	416.0 (327.0, 536.0)	0.229
	At 12 months	267.5 (253.0, 280.0)	268.0 (238.0, 283.0)	0.865
SFCT (µm)	At baseline	258.50 ± 31.89	268.33 ± 40.20	0.382
	At 12 months	231.50 ± 31.68	233.67 ± 38.79	0.842
BCVA (LogMAR)	At baseline	0.45 (0.20, 0.70)	0.45 (0.30, 0.70)	0.457
	≤ 0.3 at baseline	8 (44.44%)	11 (36.67%)	0.594
	> 0.5 at baseline	6 (33.33%)	11 (36.67%)	0.815
	At 12 months	0.30 (0.10, 0.40)	0.40 (0.30, 0.70)	0.044
	≤ 0.3 at 12 months	12 (66.67%)	14 (46.67%)	0.178
	> 0.5 at 12 months	2 (11.11%)	9 (30.00%)	0.132
Required doses of IVB in 12 months		2.00 (1.00, 3.00)	3.00 (2.00, 4.00)	0.045
Required duration of IVB treatment	≥ 6 months	8 (44.44%)	23 (76.67%)	0.024
	≥ 12 months	5 (27.78%)	17 (56.67%)	0.052

Values are expressed as the mean ± SD or median with interquartile ranges, as appropriate.

BRVO, branch retinal vein occlusion; CST, central subfield thickness; SFCT, sub-foveal choroidal thickness; BCVA, best-corrected visual acuity; IVB, intravitreal bevacizumab; ME macular edema.

Comparing the responders and poor responders, the aqueous VEGF level differed significantly (75.34 ± 84.86 vs. 95.06 ± 33.89 pg/mL, *P* = 0.046), as did the baseline CST (439.27 ± 140.04 vs. 581.67 ± 130.44 µm, *P* = 0.021). However, there was no significant difference of SFCT (sub-foveal choroidal thickness) at baseline between them. In the post hoc analyses, the proportions with grade 2 to 0 in EZD were significantly higher in the poor responders (*P* = 0.005; Table 3).

**Table 3:** Demographics and clinical characteristics of BRVO patients depending on the response to anti-VEGF treatment.

			Responsive (n = 48)	Poor response (n = 6)	*P*
Systemic factors	Sex (male:female)		16:32	1:5	0.407
	Age (years)		65.96 ± 11.73	57.67 ± 8.69	0.101
	Diabetes mellitus		15 (31.25%)	3 (50.00%)	0.358
	Hypertension		29 (60.42%)	3 (50.00%)	0.624
	Dyslipidemia		13 (27.08%)	3 (50.00%)	0.246
Aqueous humor cytokines (pg/mL)	IL-1β		0.00 (0.00;0.00)	0.00 (0.00;0.00)	0.614
	IL-2		0.20 (0.00;5.46)	19.79 (0.00;34.71)	0.087
	IL-6		5.52 (3.42;11.52)	10.67 (9.26;12.86)	0.308
	IL-8		23.70 (8.73;46.44)	34.89 (13.08;37.02)	0.650
	IL-10		0.80 (0.28;1.14)	0.46 (0.16;1.76)	0.825
	IL-17		0.00 (0.00;1.30)	0.48 (0.00;3.32)	0.408
	TNF-α		3.58 (1.91;5.45)	4.39 (2.53;5.50)	0.517
	VEGF		51.00 (29.98;83.21)	105.27 (94.33;114.66)	0.046
	PlGF		3.17 (2.13;4.00)	4.47 (3.65;6.14)	0.099
Ocular factors	Major BRVO		30 (62.50%)	5 (83.33%)	0.314
	Baseline CST (µm)		397.5 (328.0;504.5)	595.5 (447.0;634.0)	0.021
	Baseline SFCT (µm)		264.65 ± 37.26	272.00 ± 36.49	0.650
	Baseline BCVA (LogMAR)		0.45 (0.30;0.70)	0.55 (0.30;0.70)	0.900
OCT findings	Subretinal fluid		21 (43.75%)	5 (83.33%)	0.163
	EZD grade	0	27 (56.25%)	0 (0%)	0.027
		1	13 (27.08%)	3 (50.00%)	
		2	8 (16.67%)	3 (50.00%)	
	DRIL		23 (47.92%)	3 (50.00%)	1.000

Values are expressed as the mean ± SD or median and interquartile range, as appropriate.

BRVO, branch retinal vein occlusion; IL, interleukin; TNF, tumor necrosis factor; VEGF, vascular endothelial growth factor; PlGF, placental growth factor; CST, central subfield thickness; SFCT, sub-foveal choroidal thickness; BCVA, best-corrected visual acuity; EZD, ellipsoid zone disruption; DRIL Disorganization of retinal inner layers.

## Discussion

Analyzing the characteristics of BRVO according to the anatomical location, we found that the aqueous profiles and some OCT findings differed at baseline. Comparing the clinical outcomes, major BRVO required more injections in 12 months and had a worse BCVA at 12 months. The proportion of patients requiring treatment for more than 6 months was significantly higher in the major BRVO group. The poor-response group had significantly higher aqueous VEGF concentrations, CST, and proportions of grade 2 EZD at baseline.

Predicting visual function and recurrence of ME is important when deciding the treatment agent and check-up intervals in BRVO. Studies have identified factors associated with the prognosis or recurrence of BRVO using OCT and OCTA or clinical characteristics^8,10–12^. EZD is associated with the degree of ischemia or prognosis in BRVO^9,13^. A recent machine-learning study reported that retinal structure integrity as observed via OCT was associated with the visual prognosis for the treatment of BRVO^14^. Some studies have reported that the initial CST is a negative predictor of visual function and associated with recurrence in BRVO treatments^9,15,16^. In our study, a higher proportion of disintegrity of the EZ junction was seen in major BRVO patients and poor responders. The poor responders to anti-VEGF treatments also had a greater CST.

Major BRVO had poor outcomes in our series. It required longer treatment and more treatments in the first year. While the baseline BCVA did not differ significantly, the BCVA of the major BRVO group became significantly worse compared to the macular BRVO group. Studies have examined prognosis depending on the type of BRVO^17,18^. Classifying BRVO into major and macular BRVO is the easiest, most definitive way to classify BRVO according to the degree of ischemia, but few papers have examined prognosis, and most studies had few subjects^17,19^. Although many devices have been developed to assess the degree of ischemia, we feel that major versus macular BRVO is a good classification method that can predict the prognosis after a simple fundus examination. We infer that degree of ischemia affect the aqueous profiles, status of DRIL in the OCT finding, and frequency or duration of treatments.

We assessed the use of aqueous cytokines and VEGF as biomarkers for predicting the prognosis. In our series, major BRVO had significantly higher aqueous inflammatory cytokine and VEGF levels compared to macular BRVO. Patients who responded poorly to the initial anti-VEGF agents had higher aqueous VEGF levels. When the patients were divided into two groups based on the median aqueous VEGF level, the group with the higher level required more treatments, including intravitreal dexamethasone implants, in the first year (3.41 ± 1.47 vs. 2.56 ± 1.12, *P* = 0.036). Based on these findings, we infer that the aqueous humor reflects the retinal status and could be a good biomarker. Although aqueous humor is not easily available and the availability varies depending on the patient, more data could produce meaningful results.

Some previous studies used SFCT as a predictive biomarker for BRVO treatments. One study reported that the mean SFCT of eyes with a ME in the responsive group is significantly thicker than that in the refractory group^20^. They assumed that the increase in SFCT was related to an increase in intraocular VEGF concentrations. Another report showed thicker baseline SFCT is associated with better visual prognosis^21^. However, there is another conflicting report that there is no association of initial SFCT and treatments outcome^22^. In our study, there is no association between SFCT and treatments outcome. In addition, SFCT did not showed significant correlations with any aqueous VEGF or cytokines. This result might be due to differences in efficacy or molecular size between bevacizumab and ranibizumab. More studies should be conducted considering the small number of samples in previous studies including ours. Some papers have reported that baseline BCVA is good prognostic factor^10,23,24^. However, the average age is high in BRVO compared to other retinal vascular diseases and most patients diagnosed with BRVO have cataracts. We excluded patients who had undergone cataract surgery from the analysis comparing aqueous cytokine levels. After grouping BRVO patients according to logMAR BCVA ≤ 0.3 at baseline, a better BCVA at baseline was also associated with a better BCVA at the 12-month check-up (0.21 ± 0.14 vs. 0.55 ± 0.35 *P* < 0.001). However, the improvement was significantly greater in the group with worse BCVA at baseline (− 0.15 ± 0.34 vs. − 0.03 ± 0.13, *P* = 0.048) and there were no significant differences between them in the number of treatments required (2.97 ± 1.36 vs. 3.00 ± 1.41, *P* = 0.940). Therefore, in our series, the BCVA did not predict the prognosis of BRVO treatment.

There were some limitations to this study. First, we classified BRVO as simply major or macular according to the anatomical location of the occlusion. Although we performed fluorescein angiography or OCTA, we assumed that this classification reflects a difference in area of ischemia between the two. As the subgroups were relatively small, a larger sample size might have yielded more significant results. We plan to examine this in a prospective, controlled study.

In conclusion, major BRVO had a poor visual prognosis and required more and longer anti-VEGF treatments. Patients who responded poorly to anti-VEGF had higher aqueous VEGF levels and CST at baseline.

## Methods

The study protocol adhered to the tenets of the Declaration of Helsinki and was approved by the Institutional Review Board of the Catholic University of Korea. All participants provided informed consent for the use of their clinical records.

We enrolled eyes with treatment-naïve BRVO with ME with a CST ≥ 300 µm and a follow-up period of at least 18 months. Only one eye was enrolled randomly if both eyes met the inclusion criteria. The exclusion criteria were ME or hemorrhage due to other causes, and a history of uveitis, intraocular surgery, or laser treatments. We also excluded patients with macular diseases that could affect macular thickness, such as an epiretinal membrane and vitreomacular adhesions or traction.

All patients underwent ophthalmic examinations that included measuring the BCVA, fundus assessment, fluorescein angiography, and OCT (Cirrus High-Definition OCT; Carl Zeiss Meditec, Dublin, CA, USA). We selected seven consecutive horizontal B-scans including one scan centered on the fovea and the three scans immediately above and below it. The EZD in each of the scans was averaged, and graded as 0 when intact, 1 in cases of focal disruption ≤ 250 µm in length, and 2 in cases of disruption > 250 µm in length. The SFCT was measured at enhanced depth imaging mode. We defined the SFCT as the distance between the retinal pigment epithelium and the inner surface of the sclera. We measured it manually at the center of fovea using software provided by the OCT device. We defined the DRIL as lack of distinguishable boundaries between the ganglion cell—inner plexiform layer complex and inner nuclear layer, and between the inner nuclear layer and outer plexiform layer. Two retinal specialists (JWK and YJC) measured the EZD, SFCT independently, and the average values were utilized in the grading or analysis to avoid inter-observer variation. They also independently assessed the presence of DRIL. When there was disagreement over the existence of DRIL, they discussed to reach consensus.

**Figure 1:**
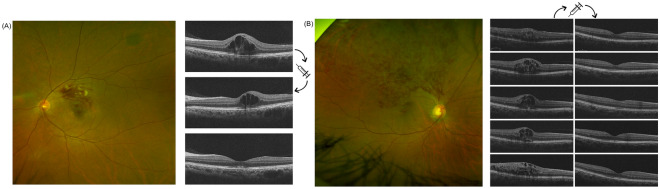
(**A**) Fundus and OCT scans of a representative patient with macular BRVO. The CST was 336 μm at baseline, and decreased to 298 μm 1 month after the first IVB, and 257 μm 2 months after the first IVB; there was no recurrence of ME that required IVB treatment. The logMAR BCVA was 0.2 at baseline, improved to 0.1 2 months after the first IVB and was maintained until 12 months. (**B**) Fundus and OCT scans of a representative patient with major BRVO. At 1 month after the initial IVB, the CST was reduced; four more IVB treatments were required in the first year. The logMAR BCVA was 0.4 at baseline and worsened to 0.5 at 12 months.

The BRVO was diagnosed by retina specialists based on the typical features of BRVO in the fundus examination, and fluorescein angiography evidence. The BRVO was subclassified by the site of vascular occlusion using previous definitions^3^. Occlusion of a temporal arcade vein or branch extending to the peripheral retina beyond the retinal vascular arcades was diagnosed as major BRVO and occlusion confined between the superior and inferior retinal temporal vascular arcades as macular BRVO (Fig. 1)^11^.

We also classified patients as either good or poor responders. Responsiveness was defined as CST < 300 µm during treatment. IVB was given using a *pro re nata* regimen when patients had CST ≥ 300 µm at a monthly check-up.

We administered up to three consecutive monthly injections of 1.25 mg IVB if there was no response. If these anti-VEGF injections failed to resolve the ME, we categorized the patients as poor responders and treated them with intravitreal dexamethasone.

We evaluated the clinical outcomes of patients at 12 months when the ME resolved. We compared the baseline characteristics and clinical outcomes between major and macular BRVO and the baseline characteristics between responders and poor responders.

### Cytokine and growth factor measurements

We measured the concentrations of IL-1β, IL-2, IL-6, IL-8, IL-10, and IL-17, TNF-α, PlGF, and VEGF, in 75-µL samples of aqueous humor. The corresponding antibodies were immobilized on beads and 75-μL aliquots of Calibrator Diluent RD6–52 (R&D Systems, Minneapolis, MN, USA) were added to the samples. Then the samples were incubated for 2 h after adding beads, for 1 h after adding detection antibodies, and for 30 min after adding streptavidin–phycoerythrin reagent. Samples were analyzed using a Luminex xMAP system (Luminex, Austin, TX, USA). All values below the lower detection limit were considered zero values.

### Statistical evaluation

The statistical analyses were performed using SPSS Statistics for Windows, ver. 21.0 (SPSS, Chicago, IL, USA). The Student’s *t*-test, Mann–Whitney *U-*test, and chi-square test were used to compare values or proportions of patient subgroups.

## Data Availability

The datasets generated during and/or analyzed during the current study are available from the corresponding author on reasonable request.
